# Role of miRNA-182 and miRNA-187 as potential biomarkers in prostate cancer and its correlation with the staging of prostate cancer

**DOI:** 10.1590/S1677-5538.IBJU.2019.0409

**Published:** 2020-03-17

**Authors:** Brusabhanu Nayak, Naveed Khan, Harshit Garg, Yashika Rustagi, Prabhjot Singh, Amlesh Seth, Amit Kumar Dinda, Seema Kaushal

**Affiliations:** 1 Department of Urology All India Institute of Medical Sciences New Delhi India Department of Urology, All India Institute of Medical Sciences, New Delhi, India;; 2 Department of Pathology All India Institute of Medical Sciences New Delhi India Department of Pathology, All India Institute of Medical Sciences, New Delhi, India

**Keywords:** MicroRNAs, Prostatic Neoplasms, Biomarkers

## Abstract

**Purpose:**

The microRNAs expression has emerged as a potential biomarker for the diagnosis and prognosis of prostate cancer. This study investigated the expression of miRNA-182 and miRNA-187 in prostate cancer patients and established a correlation between miRNA expression and staging of prostate cancer.

**Materials and Methods:**

This prospective observational study involved patients undergoing transrectal ultrasound-guided biopsy for suspicion of prostate cancer. Pre-biopsy urine samples and prostatic core tissue samples of the patients were preserved and the miRNA-182 and miRNA-187 were studied.

**Results:**

Sixty-three patients were included in this study, thirty-three patients were diagnosed with prostate cancer and thirty patients having benign histopathology were considered as controls. The expression of miRNA-182 was significantly increased (p=0.002) and miRNA-187 significantly decreased (p <0.001) in prostate cancer tissue specimens. However, the expression of these miRNAs did not significantly differ in the urine of prostate cancer patients as compared to controls. Serum Prostatic Specific Antigen (PSA) inversely correlated with the median expression of miR-187 in prostatic tissue (p=0.002). Further, the expression of miRNA-187 in prostate cancer tissue was significantly decreased in metastatic prostate cancer (p=0.037). Using ROC analysis, miRNA-187 expression was able to distinguish the presence or absence of bone metastasis [area under ROC (AUROC) (±SD) was 0.873±0.061, p <0.001].

**Conclusion:**

The miRNA-182 and miRNA-187 appear to be promising biomarkers in prostate cancer and miRNA-187 can serve as an important diagnostic marker of metastatic prostate cancer.

## INTRODUCTION

Prostate cancer is among the common cancers of men and a major cause of cancer death. The presentation of prostate cancer may vary from an indolent disease to aggressive and metastatic disease. The current recommendations for prostate cancer screening emphasize informed decision making regarding its screening in men aged 55-69 years ( 1 ). Serum Prostate Specific Antigen (PSA) has played a key role in prostate cancer, being used not only for screening but also for the diagnosis, prognosis, and follow-up of the treatment. Though serum PSA is widely used in clinical practice, a multitude of genomic markers has revolutionized the screening and management of prostate cancer ( 2 ). This has also led to an increased interest in the role of microRNAs (miRNAs) for the diagnosis of prostate cancer. The miRNAs are relatively small non-coding RNA molecules involved in cell development, differentiation, apoptosis, and cell proliferation. They can act as oncogenes and tumor suppressor genes, they are aberrantly expressed in human malignancies and play an important role in initiation, promotion, and metastases of these malignancies including prostate cancer ( 3 ). MicroRNA in the urine, serum and prostatic tissue has emerged as a potential biomarker for prostate cancer diagnosis and staging. However, few studies have addressed the role of miRNA as a potential biomarker in prostate cancer, thus limiting its clinical utility ( 4 ). Up-regulation of miRNA-182 and downregulation of miRNA-187 is associated with clinicopathological staging and progression of prostate cancers ( 5 , 6 ). In this study, we evaluated the significance of urinary and tissue microRNAs (miRNA-182 and miRNA-187) in prostate cancer patients and their association with prostate cancer staging.

## MATERIALS AND METHODS

### Study design

This prospective observational study involved patients undergoing the 12-core transrectal ultrasound (TRUS) guided prostate biopsy for evaluation of prostate cancer over a period of two years. The study was approved by Institute Ethics Committee. All patients having a suspicious prostate (from digital rectal examination) or having serum PSA levels >4ng/mL underwent TRUS guided prostate biopsy. Bone scan was done in patients with histopathological proven prostate cancer.

After informed consent, the demographic profile, serum PSA and imaging findings of the patients were noted. An additional core of prostate tissue from the suspicious area was taken during the biopsy and preserved at -80ºC. Urine samples of the same patients were taken in a 50mL sterile vial and preserved at -80^S^C before the biopsy or digital rectal examination.

### Laboratory technique for expression of miRNA 182 and miRNA 187

The miRNAs were isolated from the preserved core of tissue using a mirVana RNA Isolation Kit®. The integrity of the extracted RNA pool was checked on 1X MOPS-formaldehyde agarose gel. Low molecular weight RNA was extracted using Ambion mirVana miRNA Isolation Kit® as per the manual instructions. The RNA concentration and purity were determined spectrometrically by measuring the A260/A280 ratio using the NanoDrop ND-1000 spectrophotometer (Nanodrop Technologies). RNA samples were stored at -80°C until further use.

The details of PCR primers for miRNA 182 and miRNA187 have been provided in Supplementary Table-1. miRNAs concentrations of each sample were quantified using NanoDrop ND-1000 spectrophotometer (Nanodrop Technologies). 500ng/ul of miRNA was used as template for cDNA synthesis using Taqman microRNA Reverse transcription kit® (cat no. 4366597, Thermofisher Scientific Inc, USA).

The RNA isolation from fresh whole urine collected (50mL) involved centrifugation at 2500g, 23^o^C, for 15 minutes. The supernatant was then discarded and the remaining sediment was resuspended in 1mL of 1X PBS and again centrifuged at 2500g, 23^o^C, for 15 minutes to wash the sediments. Final pellet was used for RNA isolation with RNeasy Mini Kit® according to manufacturer’s protocol (Kit, Qiagen RNeasy).

We used microRNA isolation and purification kit (Cat No. 29000) from Norgen Biotek corp, Canada. We followed manufacturer instructions. The protocol was started with 15mL urine sample of each patient and lysis was done in lysis buffer with β-mercaptoethanol and vortexed to lyse cells. Molecular grade 99.9% ethanol was then added to precipitate miRNAs and the samples were centrifuged through a kit-supplied spin column. When all material mix was passed through, the column was then washed using the supplied wash buffer, dried and the kit elution solution applied. The final RNA was eluted in 50µL volume.

Expression levels of the miRNAs hsa-mir-182 and hsa-mir-187 were determined by quantitative real-time PCR (BIORAD C96f Real-Time PCR machine) using TaqMan microRNA Reverse transcription kit® and SoS Eva Green qPCR Master Mixes® (Biorad®) with the designed primers (Sigma®). The primers designed used are mentioned in Supplementary Table-1. The Real-Time detection of amplified PCR products was based on the detection of fluorescent signals generated by binding of SOoS Eva Green to double-stranded DNA. The fluorescent signal from each PCR reaction was collected as the peak-normalized values plotted versus the cycle numbers. The reactions were characterized by comparing the threshold cycle (Ct) values. Ct is a unitless value defined as the fractional cycle number at which the normalized sample fluorescence signal passes a fixed threshold above baseline when it is always located within the linear phase of amplification. The samples with a high starting copy number of cDNA show an increase in fluorescence earlier in the PCR process, therefore resulting in a low Ct number. The small nuclear RNA U6 served as an internal control (RNU6B). The reactions were performed in two cyclic programs at 95ºC for 30 sec, followed by 40 cycles of 95ºC for 15 sec and 55ºC for 30 sec. All reactions were run in duplicates.

### Statistical analysis

The miRNA expression of the patients along with demographic characteristics and histopathological reports were entered in a Microsoft Excel® spreadsheet. Continuous variables were expressed as mean±standard deviation (SD) or median (Interquartile range [IQR]) as appropriate. Categorical variables were compared using the chi-square test and continuous variables were compared using Wilcoxon-rank sum test and Kruskal-Wallis rank test as appropriate. Statistical significance was taken as p <0.05. Data were analyzed using IBM SPSS Statistics® software (version 20.0, Chicago. IL, USA).

## RESULTS

63 patients were included in the study. 33 patients were diagnosed with prostate cancer while the remaining 30, having no evidence of malignancy in TRUS guided biopsy, were included as controls. The baseline characteristics of the study population are presented in Table-1 .

**Table 1:** Baseline characteristics of the study population.

Parameter	Total population (n=63)	Patients with prostate cancer (n=33)	Controls (n=30)
**Mean age (±SD), years**	65.3±8.0	65.2±7.8	65.3±8.5
**Digital rectal examination**			
Firm non nodular prostatomegaly	41 (65.1%)	11 (33.3%)	30 (100%)
Hard or nodular prostatomegaly	22 (34.9%)	22(66.7%)	-
**Median PSA (IQR), ng/mL**	15 (8-50)	47 (15-100)	10 (8-14)
**Number of patients with PSA range, n (%)**			
0-10 ng/mL	21 (33.4%)	5 (15.2%)	16 (53.3%)
10-20 ng/mL	15 (23.8%)	7 (21.2%)	8 (26.7%)
>20 ng/mL	27 (42.8%)	21 (63.6%)	6 (20%)
**Histopathological Grade Group, n (%)**			
Gleason Grade group 1 (GS=6)		6 (18.1%)	
Gleason Grade group 2 (GS=3+4)		2 (6.1%)	
Gleason Grade group 3 (GS=4+3)		5(15.2%)	
Gleason Grade group 4 (GS=4+4)		5 (15.2%)	
Gleason Grade group 5 (GS=9,10)		15 (45.4%)	
**Metastasis on Bone scan, n (%)**			
Absent		16 (48.5%)	
Present		17 (51.5%)	
Oligometastatic disease (≤4 sites)		7/17 (41.2%)	

### Expression of miRNA

Two microRNAs, miR-182 and miR-187, were studied and their expression was analyzed in both tissue and urine samples. The expression of miR-182 was significantly higher (p=0.002) and miR-187 was significantly lower (p=0.001) in prostate cancer tissues as compared to controls. A similar trend was seen in urine samples but it did not reach the statistical significance level [miR-182: p=0.879 and miR-187: p=0.201). Table-2 describes the detailed expression of the various miRNA in the two groups.

**Table 2:** Median expression of miRNA-182 and miRNA-187 in patients with prostate cancer and controls.

Sample	miRNA	Prostate cancer (n=33)	Control (n=30)	p- value
Prostatic tissue	miRNA-182	4.99 (1.36, 7.58)	3.17 (0.10, 8.34)	0.002*
Prostatic tissue	miRNA-187	1.67 (1.31, 2.28)	4.60(.11, 10.51)	<0.001*
Urine	miRNA-182	4.35 (1.06, 9.89)	3.81 (.20, 7.7)	0.200
Urine	miRNA=187	1.87 (.31, 7.04)	2.11 (.10, 4.86)	0.879

### Relationship of miRNA with PSA, grade, and metastasis in prostate cancer

The patients were grouped into 3 groups based on serum PSA levels: 0-10ng/mL, 10-20ng/mL and >20ng/mL. The miRNA expressions between the various groups were compared using Kruskal-Wallis test. The miRNA expression varied inversely with increasing PSA risk category (p=0.002). However, no other significant association was observed between miRNAs expression in prostatic tissue or urine and serum PSA levels ( Table-3 ).

**Table 3:** Correlation of miRNA expression with various aspects of prostate cancer (n=33).

Parameter	Tissue miRNA-182 expression	Tissue miRNA-187 expression	Urinary miRNA-182 expression	Urinary miRNA- 187 expression
Serum PSA	0.953	0.002*	0.678	0.157
Gleason Grade group	0.841	0.567	0.879	0.721
D’Amico Risk stratification	0.547	0.066	0.547	0.212
Metastasis on bone scan	0.130	<0.001*	0.800	0.879

**PSA** = Prostate Specific Antigen; p-value calculated using Wilcoxon-rank sum test or Kruskal-Wallis test as appropriate; * p<0.05 considered as significant

Patients were divided into groups based on Gleason’s score on histopathology. Group 1 included Gleason Score <6, Group 2 included Gleason Score 3+4=7, Gleason Group 3 included Gleason Score 4+3=7, Gleason Group 4 included Gleason Score 4+4=8 and Gleason Group 5 included Gleason Score 9 or 10. The mi RNA 182 and 187 expressions did not vary significantly between these groups, based on the Kruskal-Wallis test, as shown in Table-3 . Similarly, miRNA expressions did not vary significantly between various classes of D’Amico risk stratification of prostate cancer.

**Figure 1:**
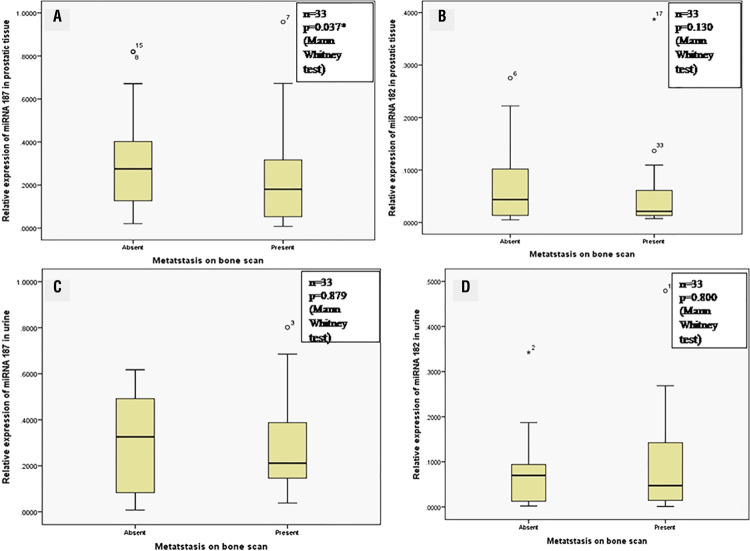
Graph depicting the association between presence or absence of bone metastases with A) miRNA-187 expression in prostatic tissue; B) miRNA-182 expression in prostatic tissue; C) miRNA-187 expression in urine; D) miRNA-182 expression in urine in prostate cancer patients.

Bone scan was done on patients with prostate cancer. 17 patients had metastases while 16 patients did not have metastasis. The expression of miR-187 was significantly decreased in prostate biopsy of metastatic prostate cancer patients (p <0.001, Kruskal Wallis test). However, there was no significant difference between miR-182 expression in prostatic biopsy tissues and miRNA expression in urine with metastases. Figure-1 depicts the correlation of expression of various miRNAs with the presence or absence of metastases.

**Figure 2:**
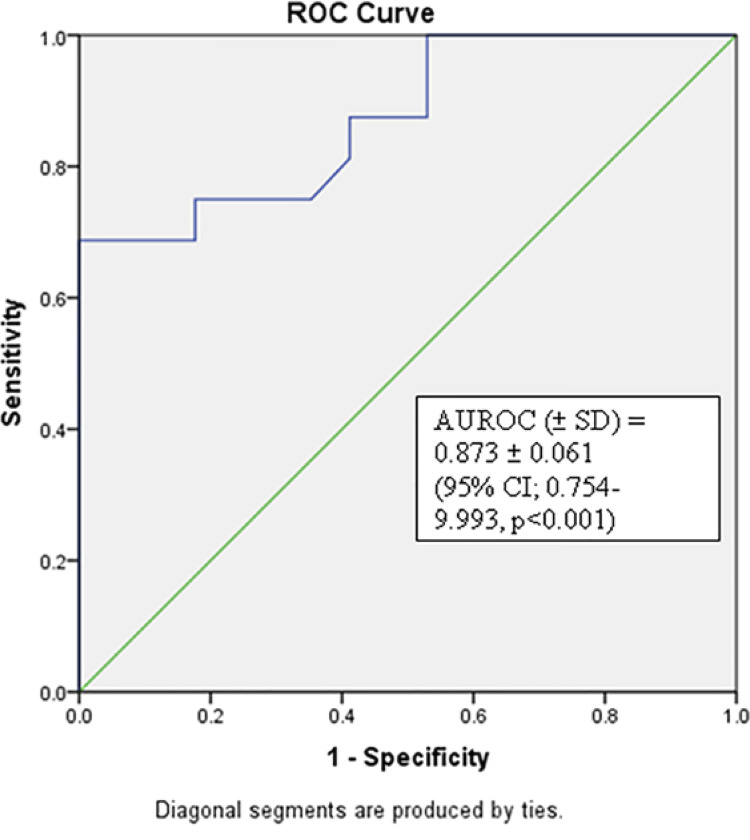
Receiver operating characteristic curve of mi RNA-187 expression in prostatic tissue with presence or absence of metastasis on bone scan;

Using ROC analysis to study the utility of miR-187 expression to distinguish the presence or absence of bone metastasis, area under ROC (AUROC) (±SD) was 0.873±0.061 (95% CI; 0.754-9.993, p <0.001). Using Youden’s index method, the median expression of miR-187 in prostatic tissue of 2.00 had 68.8% sensitivity and 100% specificity to predict the presence of bone metastases in prostate cancer ( Figure-2 ).

## DISCUSSION

The miRNAs play an important role on cellular differentiation including the biochemical signalling of various oncogenic pathways ( 7 , 8 ). The miRNAs alter the cell cycle regulation, angiogenesis, and metastasis but the deciphering the exact relation between miRNA and cancer is complex ( 9 - 11 ). In malignancy, the differential expression of miRNA appears to be the cause as well as the effect of oncogenesis. The miRNAs can be both oncogenic or anti-oncogenic ( 12 - 14 ). Thus, although the differential expression may provide diagnostic and prognostic benefit, the actual reason for such a change is multi-factorial and still to be deciphered.

Over the last few years, various studies have identified miRNAs that are differentially expressed in prostate cancer. The expressions of these miRNAs have been linked to androgen signaling as well as clinic-pathological factors ( 15 - 17 ). Furthermore, it has been advocated that miRNAs may be new contenders for cancer drug treatment, given the oncogenic or tumor suppressive functions of miRNAs ( 18 ). Nevertheless, the results of the various studies are conflicting.

Detection of clinically significant prostate cancer and identification of the suitable candidates for active surveillance versus radical treatment forms the mainstay of management of prostate cancer. At present, PSA kinetics, tumor grade (Gleason score), and the clinical stage classify the prostate cancer patients. Even though these factors are clinically beneficial, they have limitations in identifying cases, predicting disease outcomes and controlling clinical management decisions ( 19 - 21 ). Thus, new biomarkers are needed to improve existing diagnostic, prognostic and treatment management strategies.

We investigated the abnormal expression of miRNAs based on expression signatures in prostate cancer. Upregulation of miR-182 was formerly described in prostate cancer and other tumors, while miR-187 was later found to be lost in prostate cancer and ovarian carcinoma but overexpressed in breast cancer progression ( 22 - 25 ).

In this study, we found the upregulation of miR-182 and downregulation of miR-187 in prostate cancer. Similar results were reported by Casanova-Salas et al. ( 26 ). Furthermore, miR-182 and miR-187 were also differentially expressed according to clinical variables, such as the tumor stage, Gleason score, the status of TMPRSS2-ERG and progression. Fuse et al. ( 22 ) also reported the downregulation of miR-187 along with miR-224, 34 and 221 in prostate cancer.

In another study by Schaefer et al., ( 24 ) the miRNA expression was correlated with histopathological grade and clinical stage of prostate cancer. They identified ten microRNAs including hsa-miR-16, hsa-miR-31 etc being downregulated while 5 miRNAs including hsa-miR-182 upregulated in prostate cancer. The expression of upregulated miRNAs correlated significantly with tumor stage and grade. Moreover, two microRNAs classified up to 84% of malignant and non-malignant samples correctly. This highlighted the role of differential expression of miRNA as diagnostic and prognostic marker of prostate cancer. However, in another study by Tsuchiyama et al., ( 27 ), the expression of various miRNAs did not vary significantly among various Gleason patterns.

In this study, we did not find any statistically significant association between miR-182 expression and clinical-pathological parameters. However, we found an association between miR-187 expression and metastatic prostate cancer. We also report the role of miR-187 in diagnostic utility to differentiate the presence or absence of metastases with AUROC of 0.873 (±0.061).

Moreover, miRNA expression assessment in extracellular body fluids such as plasma, serum, saliva or urine may provide a benefit in cancer diagnosis, detection of progression and recurrence of prostate cancer. The feasibility of urine-based testing in prostate cancer has previously been documented in some studies ( 4 ).

Casanova-Salas et al. ( 28 ) studied 92 patients of prostate cancer undergoing needle biopsy, and proposed a prediction model involving miR-187, urine PCA3 and serum PSA with a sensitivity of 88.6% and specificity of 50% specificity and 69.3% diagnostic precision, which was significantly higher than PSA alone. Srivastava et al. ( 29 ) evaluated the expression of 8 miRNAs in urine and tissue samples of prostate cancer. miR-205 and miR-214 were significantly downregulated in prostate cancer patients in both tissue and urine specimens. This miRNA profile was reported to distinguish patients of prostate cancer from healthy individuals with a sensitivity of 89% and a specificity of 80%. Baumann et al. ( 30 ) studied mi-RNA 182 expression using in situ hybridization of two prostatic tissue microarrays and reported significantly higher mi-RNA 182 expression in cancer epithelium as compared to adjacent benign epithelium. However, ratio of miR-182 expression in cancer vs benign cells per patient was inversely associated with recurrence in a multivariate logistic regression model.

Haj-Ahmad et al. ( 31 ) performed miRNA expression profiling in urine samples of healthy males, BPH patients and prostate cancer patients using whole genome expression analysis. They found that the differential expression of two individual miRNAs (miR-1825 and -484) between healthy people and BPH patients was identified and found to possibly target genes related to prostate cancer development and progression among 894 miRNAs assayed. This study evaluated the expression of miRNA in urine but did not find any significant difference in prostate cancer patients.

This study has several strengths. It was a prospective study including patients with prostate cancer and the controls with a similar demographic profile. The histopathology was studied by a single genital-urinary pathologist and the expression of miRNA was done in a standardized manner. Both urine sample and tissue samples were used to study the expression of miRNA. Using ROC analysis, miR-187 appeared to have a role to distinguish the presence or absence of bone metastasis in carcinoma prostate.

However, there are certain limitations in this study. Firstly, we selected the pre-identified miRNA for this study and did not perform microarray analysis for identification of all dysregulated miRNAs. Secondly, the limited sample size may be a possible explanation for the lack of correlation between miRNA expression and clinical-pathological features. Thirdly, the lack of statistical findings might be due to unsampled tumor in the control group, especially since MRI was not performed. Fourthly, the inverse relationship between miR-187 and PSA is likely due to the fact that miR-187 tracks with cancer, not that it tracks independently with PSA. While miRNA-182 and 187 are biomarkers, they may not necessarily convey obvious function. Lastly, we did not analyze the miRNAs in serum which could have been an additional marker for prostate cancer diagnosis. Metastasis work-up using Ga-PSMA PET scan would have been a better modality as compared to the bone scan. However, in a resource-limited setup, PSMA PET was not feasible for all patients.

## CONCLUSIONS

The microRNA expression is a potential tool to improve existing diagnostic, prognostic and treatment strategies for prostate cancer. The miRNA-182 and miRNA-187 appear as important biomarkers in prostate cancer, and miRNA-187 may be used to increase the diagnostic and prognostic accuracy in the management of prostate cancer.

## APPENDIX

**Table:** Supplementary Table 1 - Details of primer sequence of miRNA-182 and miRNA 187 used for reverse transcriptase reaction.

Name of miRNA	Sequence (5’-3’) of primer
miRNA-182	ACTTTTGGCAATGGTAGAACTCAC
	GTGCAGGGTCCGAGGT
miRNA-187	TCGTGTCTTGTGTTGCAGC
	GTGCAGGGTCCGAGGT
